# Supplementary material for: Cellular 3D-reconstruction and analysis in the human cerebral cortex using automatic serial sections
Source: Commun Biol. 2021 Sep 2;4:1030. doi: 10.1038/s42003-021-02548-6 (PMC8413324; doi:10.1038/s42003-021-02548-6)
Supplement: Supplementary file 2 — Supplementary Information [file 42003_2021_2548_MOESM2_ESM.pdf]

Supplementary Note 1

Improving section quality: avoiding wrinkles

Problems in sectioning can compromise both the sample quality and the reliability of the subsequent analysis. The presence of many wrinkles on the sections can make the data analysis challenging to quantify and can potentially destroy the image dataset. Two types of wrinkles were observed during section collection on tape with the AutoCUTS-LM denoted macro-folds and micro-folds. Macro-folds deform the section and may cover a large area of the ROI. Micro-folds, on the other hand, can appear anywhere on the section. Suggestions will be given on how to prevent and minimize the effect of factors that influence the creation of wrinkles in sections during cutting.

Sample size and tissue heterogeneity

If the sections are small, they develop fewer wrinkles since they have a smaller surface where wrinkles can be generated. In **Fig.S9**, large wrinkles around excess embedding resin surrounding the tissue were generated due to density differences between the sample and the empty resin. Therefore, we recommend removing all excess resin by trimming the block as close to the sample as possible until there is only a rectangular block of embedded tissue. Nevertheless, it is challenging to remove resin around smaller non-homogeneous samples, such as C. elegans or Drosophila. For them, it is better to reduce the hardness of the resin so that the sections can be easily flattened as they are cut.

Section thickness

The quality of sections depends a lot on the cutting thickness. Ultra-thin sections (30-100 nm) generate few or no micro-folds compared to semi-thin (100-500 nm) sections. Sections with a cutting thickness above 300 nm were more susceptible to generate micro-folds compared to thinner sections when collected on tape, as shown in **Fig.S10**. Furthermore, thick sections generated more wrinkles but provided increased contrast of cells compared to thinner sections, which generated fewer micro-folds but also provided less contrast of cells.

Tape damage

Deformation on the tape, such as scratches or other damage caused by the AutoCUTS-LM reel-to-reel conveyor belt, could result in micro-folds on sections, **Fig.S12**. This problem was avoided by removing the protective coat on the section-collection side during the collection of sections. Other research groups have experienced wrinkles by using an automated tape-collecting ultramicrotome, and it could be caused by tape damage. In our case, we were using transparent tape, and it was easy to spot any damage compared to the solid coating tape used for Scanning EM.

Humidity and hydrophilicity

One of the main factors that could cause wrinkles on a section was the indoor humidity level. The tissue in **Fig.S10** was sampled with a humidity level around 10-20% because of the drying effect of the air condition in the room. If the tape's surface was exceptionally hydrophilic and the indoor environment was dry, it could cause any section to adhere without time to expand, see **Fig.S13**. Indoor temperatures above 20°C and a humidity level above 60% have a fundamental impact in decreasing the flexural and compressive strength of the epoxy. Hence, a softer resin reduces the amount of naturally generated wrinkles for semi-thin sections. We purchased a humidifier (YADU Company, China) to monitor and retain a humidity level of between 80-90% during section collections. Sections with a cutting thickness above 300 nm were more susceptible to generate wrinkles during the cutting process, as shown in **Fig.S11**. This was caused by a combination of the humidity in the room, section thickness and hydrophilicity of the tape. Nevertheless, a too high humidity level ($>$90%) can have a devastating effect on the samples. This is shown in **Fig.S14**, where some areas of the section can adhere to the tape due to the hydrophilicity and other areas might move around on the tape. This will cause the section to be unstable and generate many folds. We suggest keeping the humidity level around 85% to avoid folds.

Supplementary Tables

Table S1: Measurements and classification of identified objects for the entire stack of images for each subject. The dimensions of the observation windows (x,y,z), the total number of cells/objects, pyramidal cells, non-pyramidal cells, outliers. The density was calculated by the total number of pyramidal cells pr. volume for each subject of layer III. Excluded (%) is percentage of excluded datapoints for each subject, where Excluded (%)=(Non-pyramidal+outliers)/Total·100. The entries for the pyramidal cell density state the mean and ± one standard deviation. Mean is the average measurement at n=3, Coefficient of variation CV = SD/mean. The average for Subject 1_1 and Subject 1_2 were used for subject 1.

Brain	Window (μm)	Nr. Total	Nr. Pyramidal	Nr. Non-pyramidal	Nr. Outliers	Excluded (%)	Pyramidal cell density(mm^{-3})
Subject 1_1	635 × 1013 × 334	8872	6864	2008	0	23	31952
Subject 1_2	507 × 1001 × 511	12587	9501	3085	1	25	32569
Subject 2	488 × 1216 × 682	17534	11111	6404	19	37	27455
Subject 3	664 × 1055 × 725	16393	12586	3779	28	23	24764
Mean							28160±3101
CV							0.11

Table S2: The table shows the volume comparison between 2D vs 3D approches from the values in Table 4. Vol_{3D} is the directly measured volume based on voxel counts. Vol_L and Vol_{All} are calculated from the average segment length measured from the nucleator probe. Difference (%) is the relative change in percentage Diff $=(Vol_x - Vol_{3D})/Vol_{3D} \cdot 100$ where Vol_x is either Vol_L or Vol_{All}, Mean is the average measurement for each column, Coefficient of variation CV = SD/mean.

Brain	Vol_{3D} (μm^3)	Vol_L (μm^3)	Difference (%)	Vol_{3D} (μm^3)	Vol_{All} (μm^3)	Difference (%)
Subject 1	867	721	17	867	198	77
Subject 2	709	721	1.7	709	143	80
Subject 3	808	749	7.3	808	210	74
Mean	795 ± 65	730 ± 13	8.7 ± 6.3	795 ± 65	183 ± 29	77 ± 2.45
CV	0.08	0.18	0.72	0.08	0.06	0.03

Table S3: The table shows the volume comparison between 2D vs 3D approaches from the values in Table 4. Dia_{3D} is the equivalent diameter of an approximated sphere equal to Vol_{3D}. Dia_L and Dia_{All} are the average segment length measured from the nucleator probe. Difference (%) is the relative change in percentage Diff $=(Dia_x - Dia_{3D})/Dia_{3D}{\cdot}100$ where Dia_x is either Dia_L or Dia_{All}, Mean is the average measurement for each column, Coefficient of variation CV = SD/mean.

Brain	Dia_{3D} (μ)	Dia_L (μ)	Difference (%)	Dia_{3D} (μ)	Dia_{All} (μ)	Difference (%)
Subject 1	11.83	11.13	5.9	11.83	7.23	39
Subject 2	11.06	11.13	0.6	11.06	6.49	41
Subject 3	11.56	11.27	2.5	11.56	7.37	36
Mean	11.48 ± 0.32	11.17 ± 0.07	2.9 ± 2	11.48 ± 0.32	7.03 ± 0.39	39 ± 2
CV	0.03	0.01	0.68	0.03	0.06	0.05

Supplementary Figures

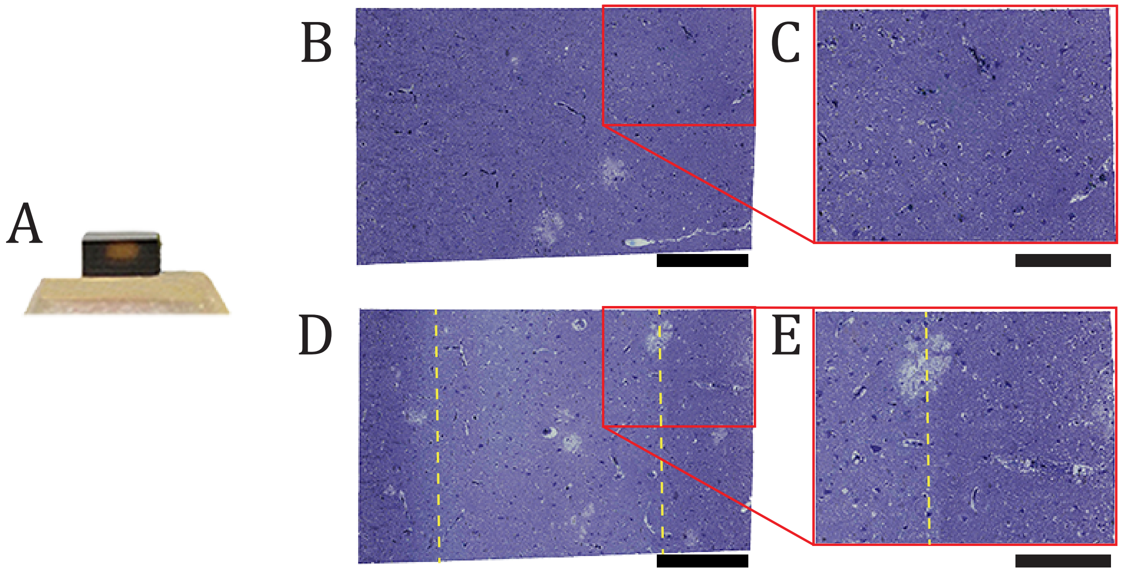

Figure S1: The influence of osmium staining and section contrast. Osmium has not entirely infiltrated the entire biopsy and gives us the ideal opportunity to test its effect on sections that need to be later stained with toluidine blue. **(A)** Biopsy stained with osmium(black color) where no osmium was present in the middle part of the block. **(B)** Biopsy section with osmium from the top part of the block stained with toluidine blue. Scale bar=400 μm. **(C)** Magnified view of the red box where the contrast between the neurons and the background is not evident. Scale bar=200 μm. **(D)** Biopsy section stained with toluidine blue from the middle part of the block, where osmium only appears in the right and left side of the section (marked with yellow lines). Scale bar=400 μm.**(E)** Magnified view of the red box for comparing the contrast between neurons and background in areas with and without osmium (right and left side of the yellow line, respectively). Scale bar=200 μm.

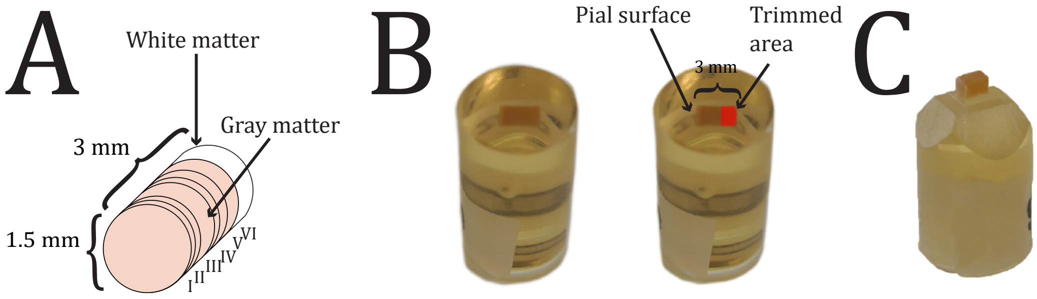

Figure S2: Sample embedding. **(A)** Illustration of the biopsy with a diameter of 1.5 *mm* and a length of ∼3 *mm*. **(B)** Sample inside the epoxy resin. The red area indicates the trimmed part of the biopsy after the ROI was located from the outermost section. **(C)** Trimmed block of sample.

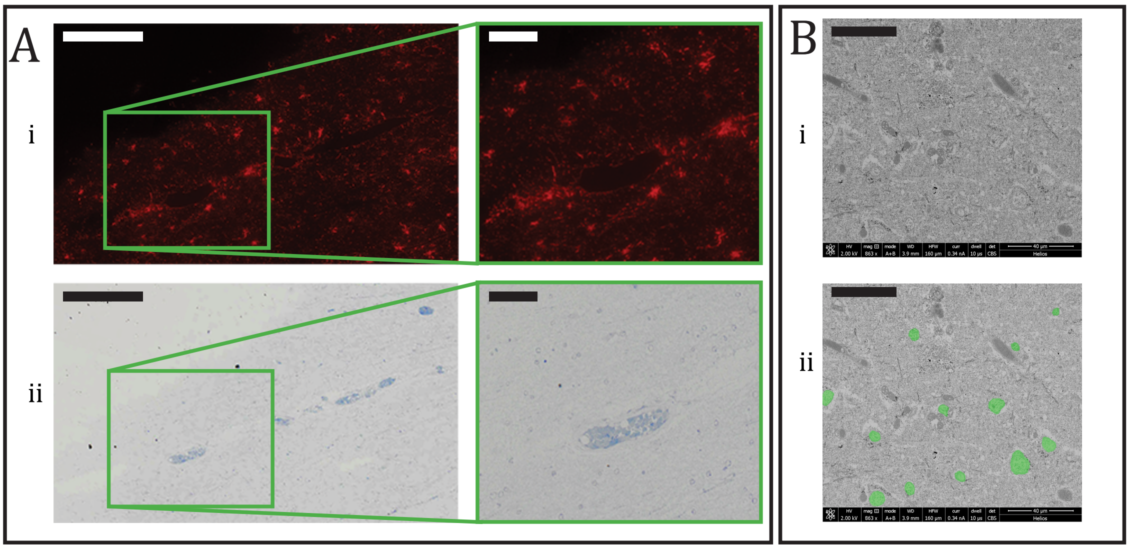

Figure S3: Light microscopy and EM imaging on biopsy from BA46. **(A)** Immunolabeled and toluidine blue-stained section. **(i)** Fluorescently immuno-label staining of cells containing glial fibrillary acid protein was observed with a red channel.**(ii)** Toluidine blue staining on the same sections. The stained vessel served as an alignment reference to combine the first and second image. Overview image: scale bar=200 μm. Zoomed in image: scale bar=50 μm. **(B)** EM imaging on a section. **(i)** Overview image with a horizontal field width (HFW) that is 160 μm. **(ii)** Neurons were manually marked with green color as an overlay on the original image. Scale bar=40 μm

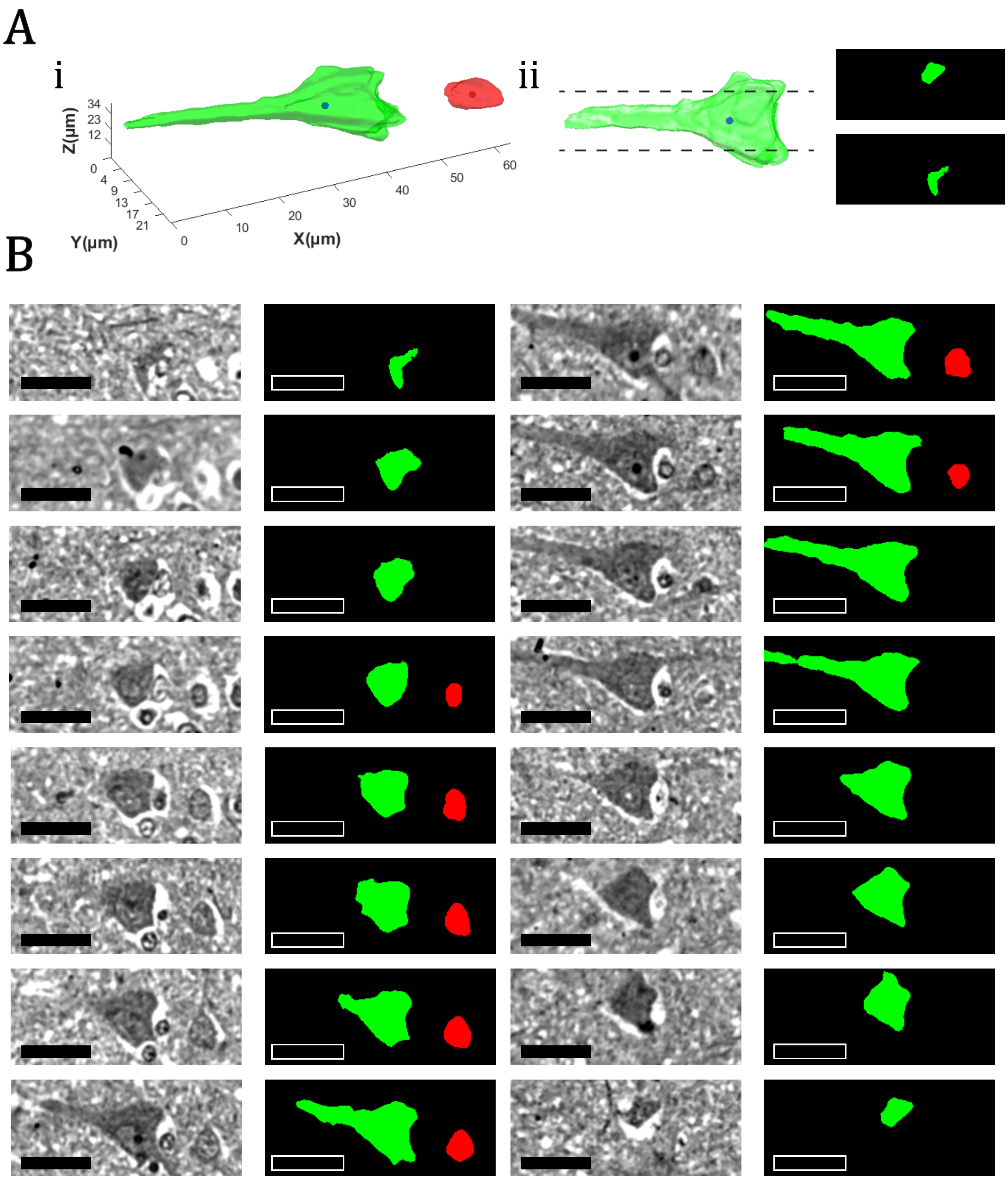

Figure S4: Demonstrate that additional cell filtering is necessary based on 3D measurements. **(A)** 3D-reconstruct of a series of image profiles from pyramidal (green) and a non-pyramidal (red) cell (i). If the reconstruction is not accomplished, single image profiles near the edge can imitate sections of smaller cells/gial cells (ii). **(B)** Pyramidal (green) and non-pyramidal (red) cell image profiles were used to create the 3D reconstruction. Scale bar=20 μm.

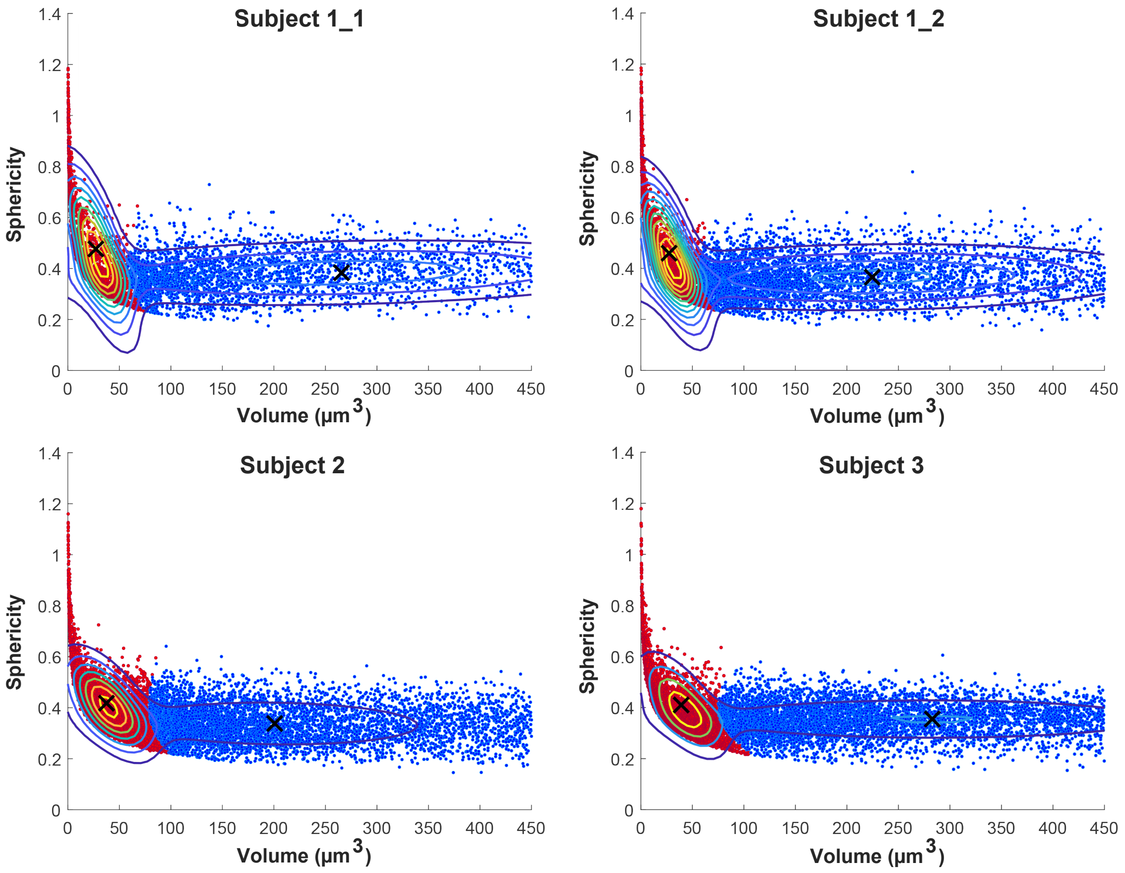

Figure S5: Displaying filtration of data. K-means clustering algorithm was performed to filter smaller neurons and artefacts from the dataset. The data was segmented into non-pyramidal and pyramidal neurons employing estimations of neuronal volume (x-axis) against sphericity (y-axis). For each subject, The data points for non-pyramidal and pyramidal cells are represented by the red and blue points, respectively.

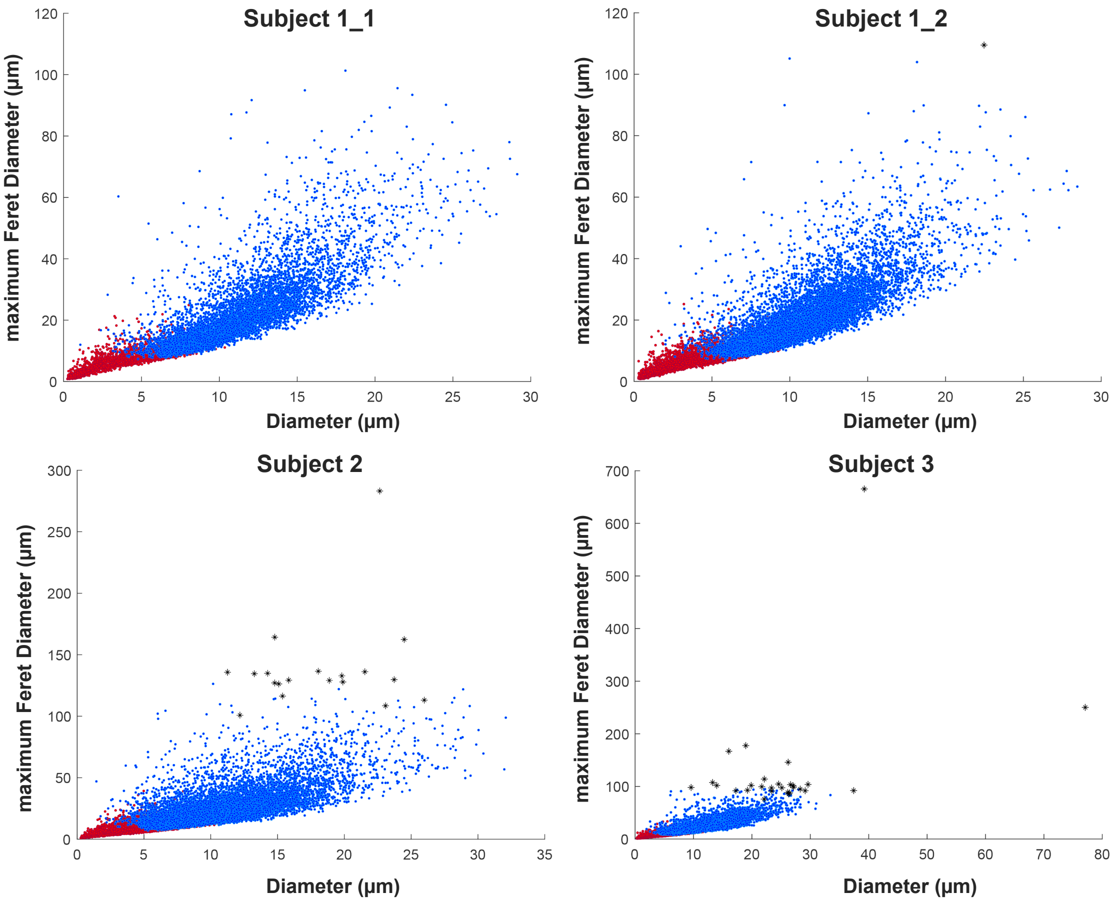

Figure S6: Displaying points for pyramidal, non-pyramidal and outliers for each subject. The graphs show plots of the largest cell profile (x-axis) against the maximum Feret Diameter in 3D (y-axis) of each neuron. For each subject, the data points for pyramidal cells are shown as blue points, while the red and black points are non-pyramidal cells, as classified with the k-means method, and outliers, respectively.

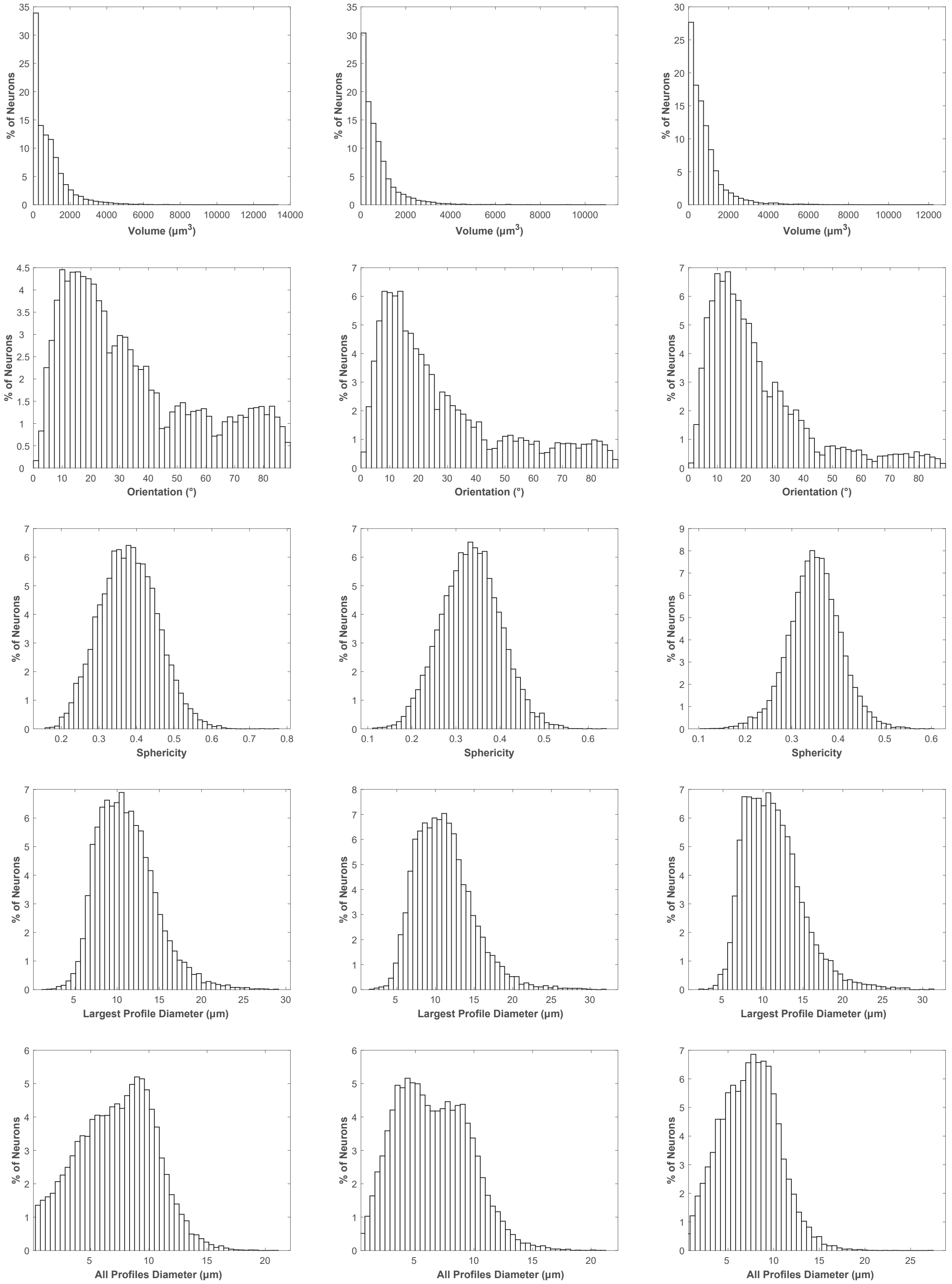

Figure S7: Quantitative measurements of pyramidal neurons. Histograms showing neuronal volume, sphericity, orientation and diameter of pyramidal cells in BA46 in layer III for each subject. The columns correspond to Subjects 1, 2, and 3, respectively.

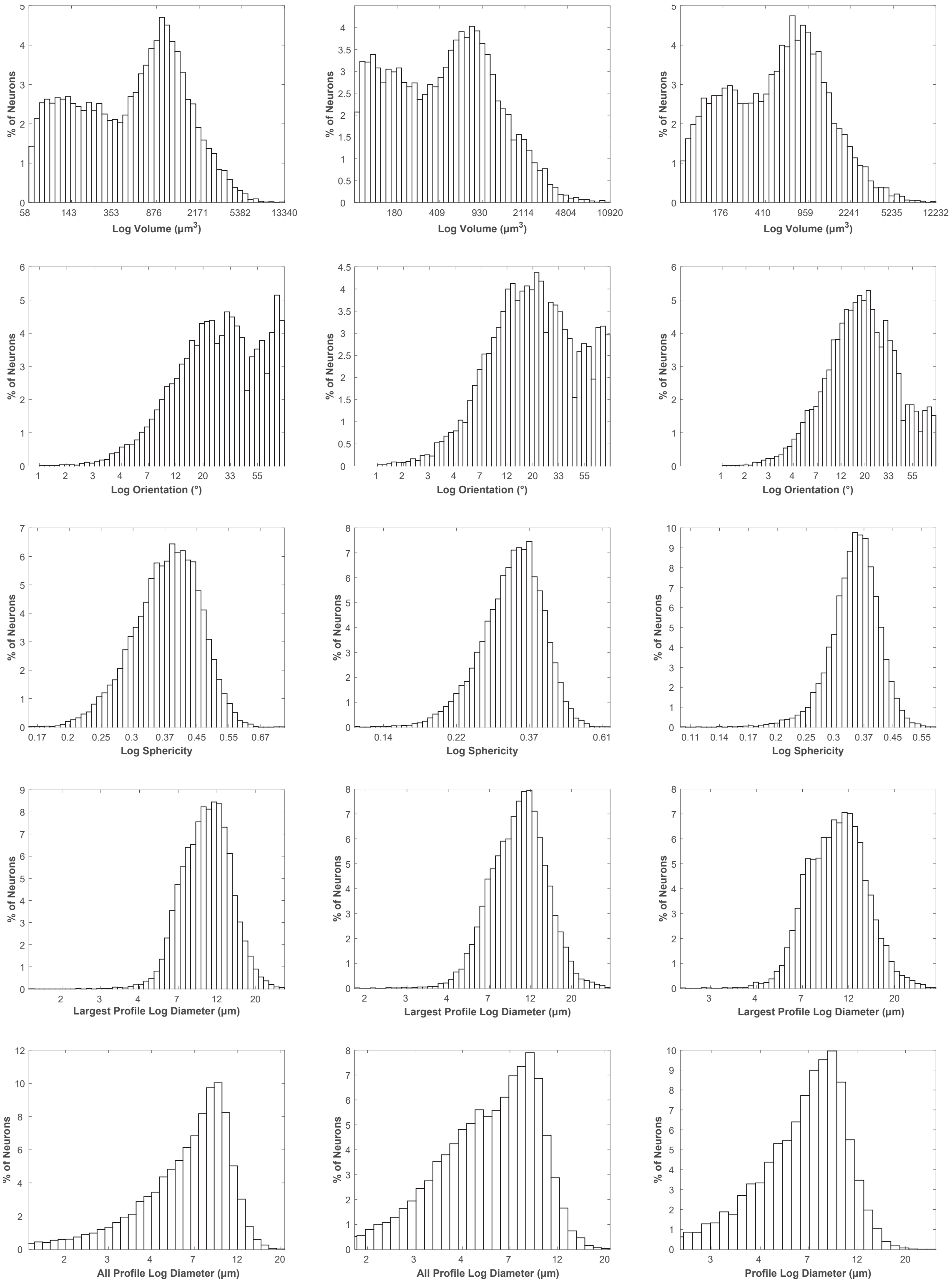

Figure S8: Quantitative measurements of pyramidal neurons log-normal transformed. Histograms showing the log-normal transformed neuronal volume, sphericity, orientation and diameter of pyramidal cells in BA46 in layer III for each subject. The columns correspond to Subjects 1, 2, and 3, respectively.

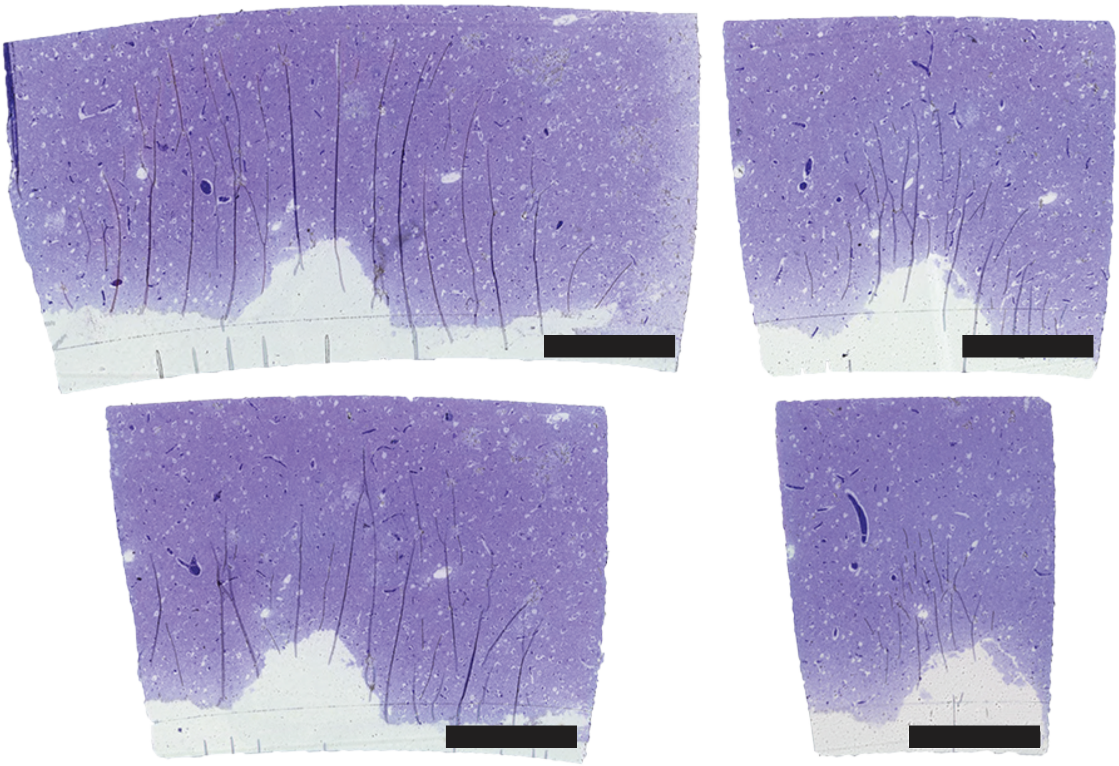

Figure S9: The connection between cutting surface density and folds. A section (2.8x1.3 *mm*) was trimmed four times to visualise the relationship between section area and macro-folds. Macro-folds will most likely be generated in more extended sections and around the blank resin area. The room temperature and indoor humidity were, 20°C and 50%, respectively. Scale bar=500 μm.

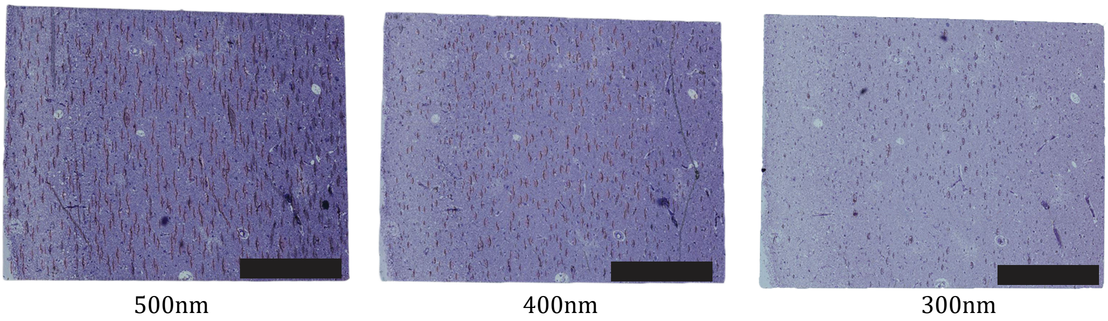

Figure S10: The connection between section thickness and folds. The number of folds and staining penetration was reduced along with the cutting thickness. The sections were stained all together with toluidine for 15 mins. The room temperature and indoor humidity were, respectively, 20°C and 10%. Scale bar=400 μm.

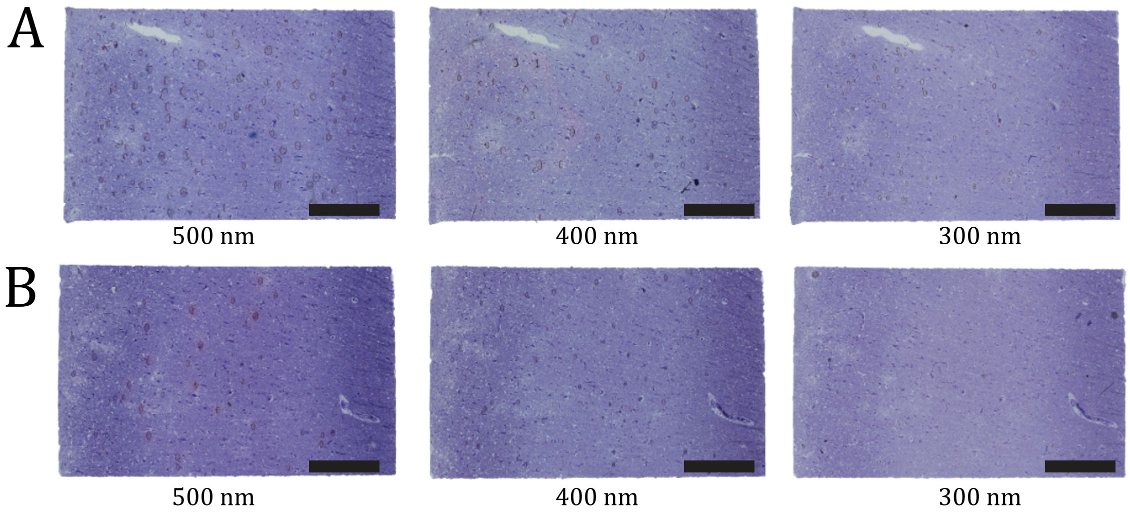

Figure S11: Test sections with a section thickness that range from 500 to 300 ***nm*** **with different indoor humidity**. **(A)** Sections were cut with an indoor humidity of 60% and thinner sections generate fewer folds. **(B)** Sections were cut with a humidity between 85-90% and there are few to none folds on the 300 *nm* thick section. All sections were stained together with toluidine blue for 15 min in room temperature at the same time. Scale bar=400 μm.

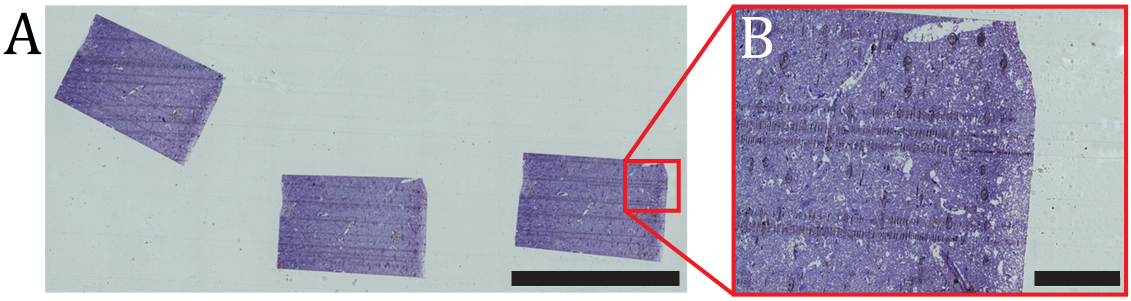

Figure S12: Damage on tape caused by AutoCUTS-LM could generate folds on the sections due to the deformation of the tape. **(A)** The damage to the tape creates pronounced lines that impact the three collected sections. Scale bar=2 *mm*.**(B)** The three visible lines were on the underside of the tape and not on the upper-side where the sections were collected. Scale bar=200 μm.

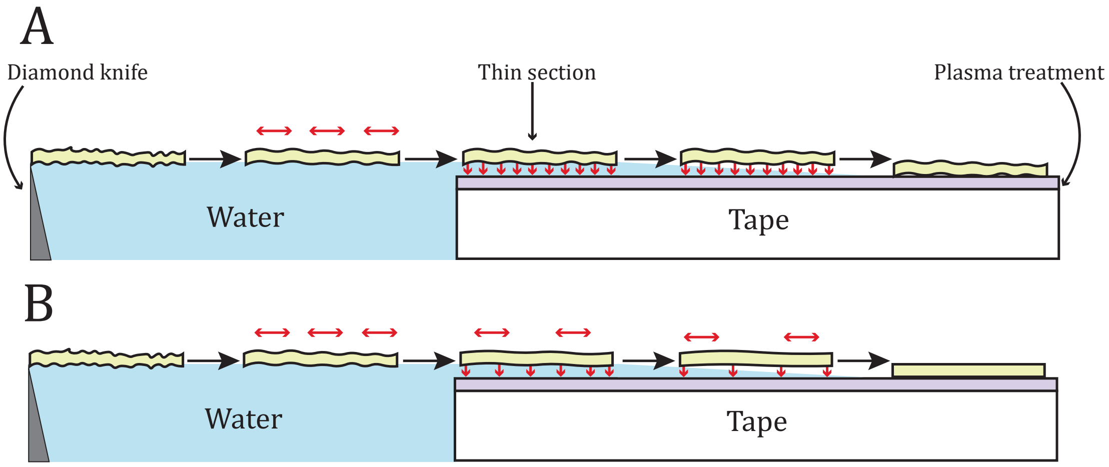

Figure S13: Illustration of section flatness caused by humidity and tape adhesion. After a section was cut with the diamond knife, it would expand in the water. **(A)** In a dry environment, sections did not have enough time to expand since they adhere to the tape almost immediately after the cutting. **(B)** Condensation on the tape surface can develop when the indoor humidity is raised, allowing the sections to expand.

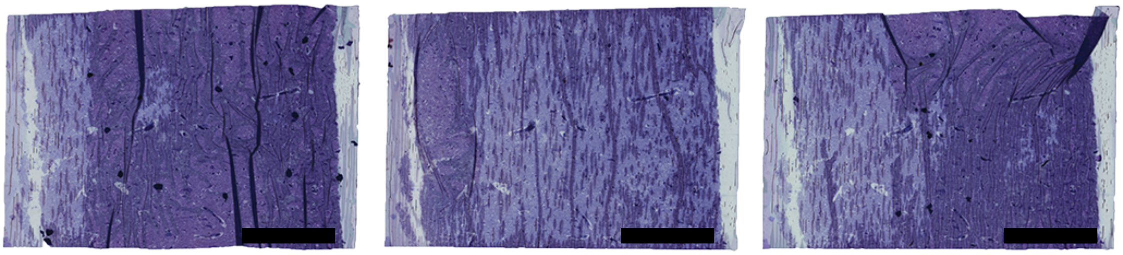

Figure S14: The impact of sampling sections with too high humidity. High humidity above 90% made the sections unstable as they fell of the collection tape. One part of the section may have been attached to the more dry sections of the tape, and some pieces of the section may have moved around. Due to the unstableness of the sections during the set, several folds were formed. Scale bar=500 μm.

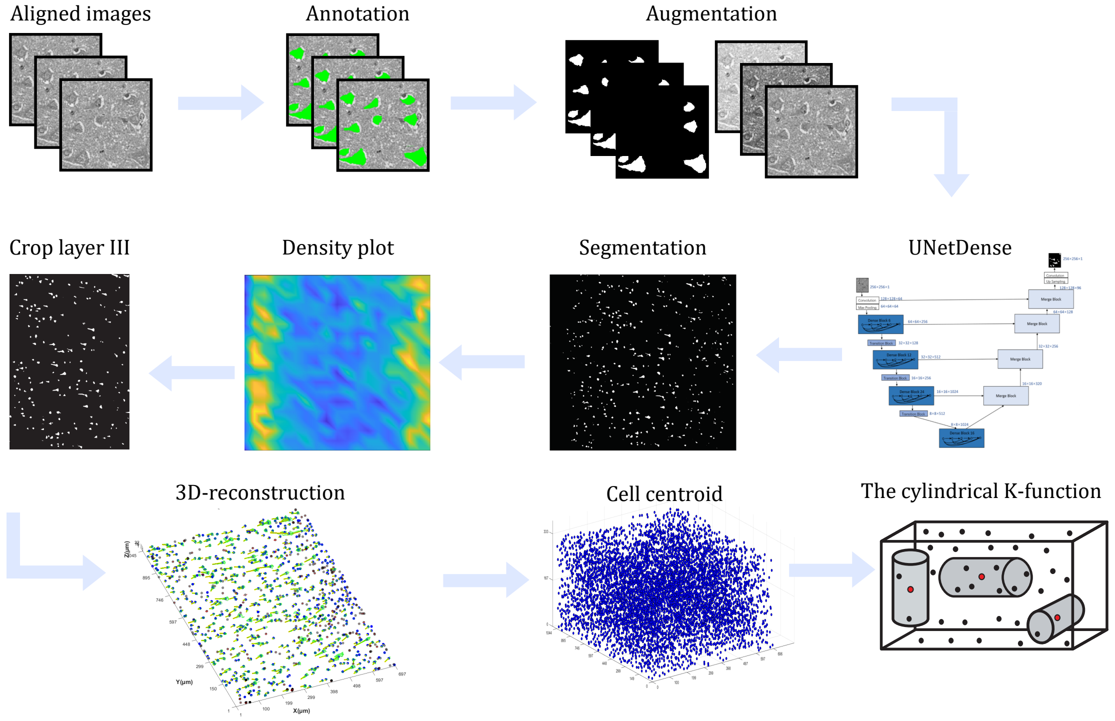

Figure S15: The data analysis pipeline for this study. First, we would manually annotate pyramidal cells from 35 images. To produce the training (5600) and validation (1400) set, each of those 35 images was subsequently augmented into 200 images with a scale of 256x256 pixels. Based on those images, the UNetDense model trained itself and segmented neurons for each subject. A density map of the centroids was used to identify the ROI and crop the stack of images. Pyramidal cell parameters were then determined based on their 3D-reconstruction, and the 3D point pattern consisting of the centroids was analyzed with the cylindrical K-function in order to detect columnar structures.

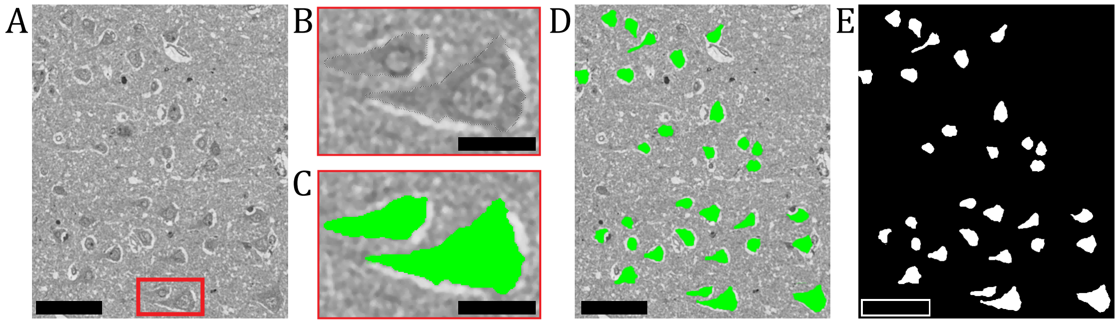

Figure S16: Manual annotation of pyramidal cells. **(A)** displays the original picture and the area we want to annotate is displayed in the red boundary frame. Scale bar=35 μm. **(B)** Photoshop's fast selection tool was used to identify the pyramidal cell annotation region. Scale bar=10 μm. **(C)** A green-colored mask was placed on top of the original image of each pyramidal cell. Scale bar=10 μm. **(D)** The annotation of cells was repeated for the rest of the image in **A** until all cells were labeled in green. Scale bar=35 μm.**(E)** mask images were translated to binary images in MATLAB where the pixel value of the annotated cells was equal to 1. Scale bar=35 μm.

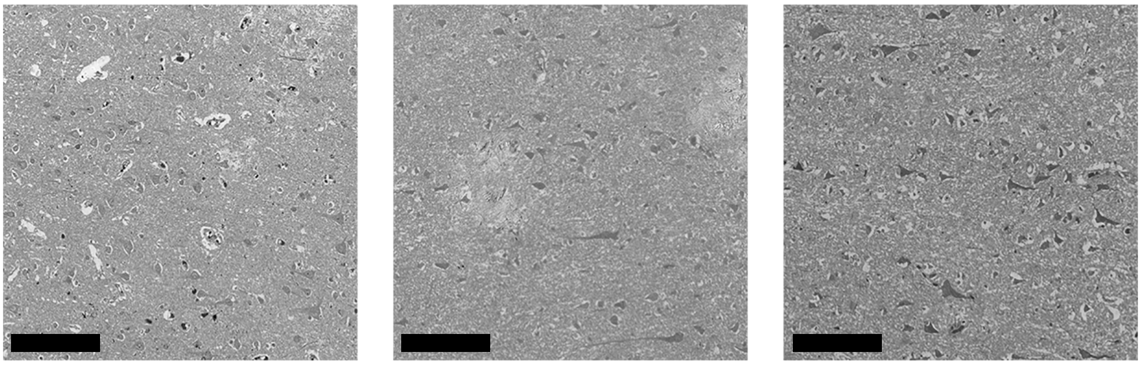

Figure S17: Validation images that were used to test the combined and individual UNetDense models. These images show the differences between sections from different subjects after staining. The images from left to right correspond to Subjects 1, 2, and 3, respectively. Scale bar=140 μm.

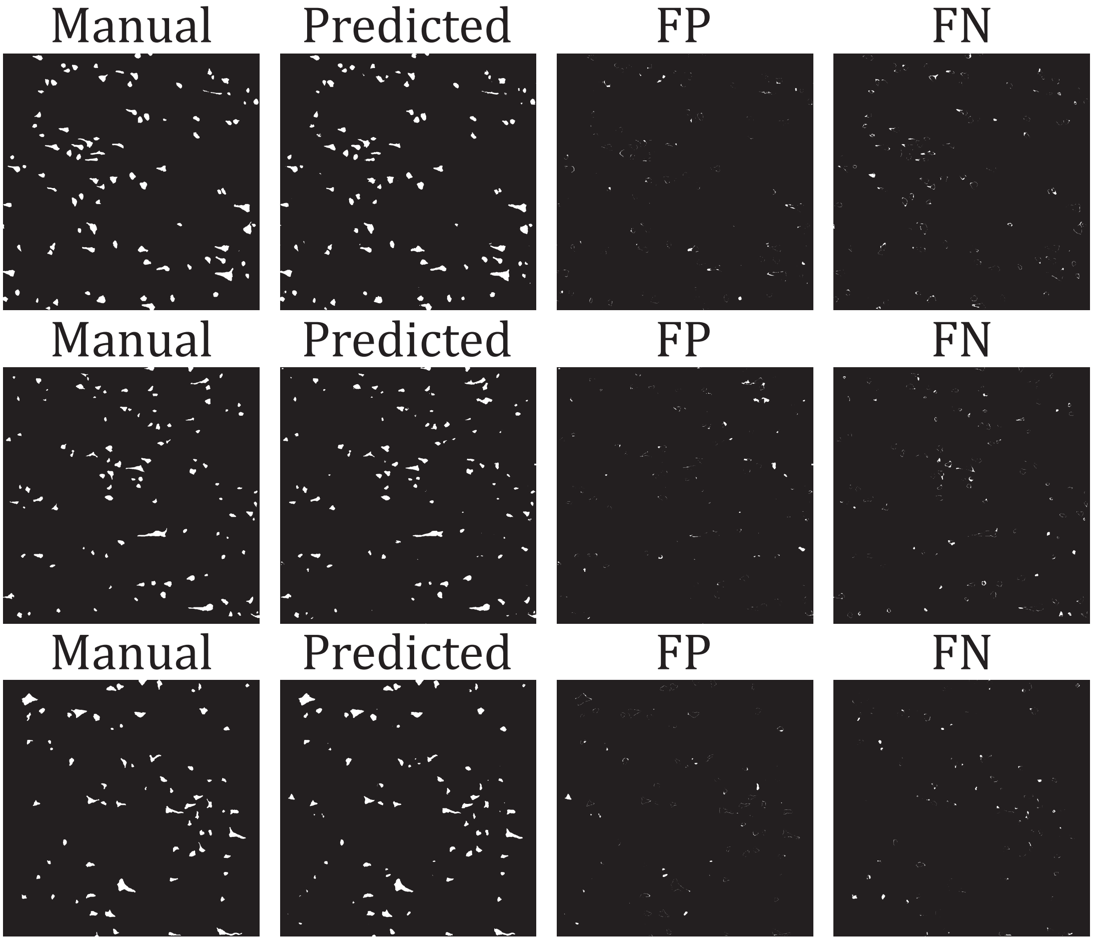

Figure S18: Test of performance of individual UNetDense models on validation images. Pyramidal cells from **Fig.S17** were manually marked by (NYL), and the same images were processed using individual UNetDense models to predict and segment pyramidal cells. The figure shows pixels which have been measured as FP, FN, and quantitative comparisons can be found in **Table 1**. The rows correspond to Subjects 1, 2, and 3, respectively.

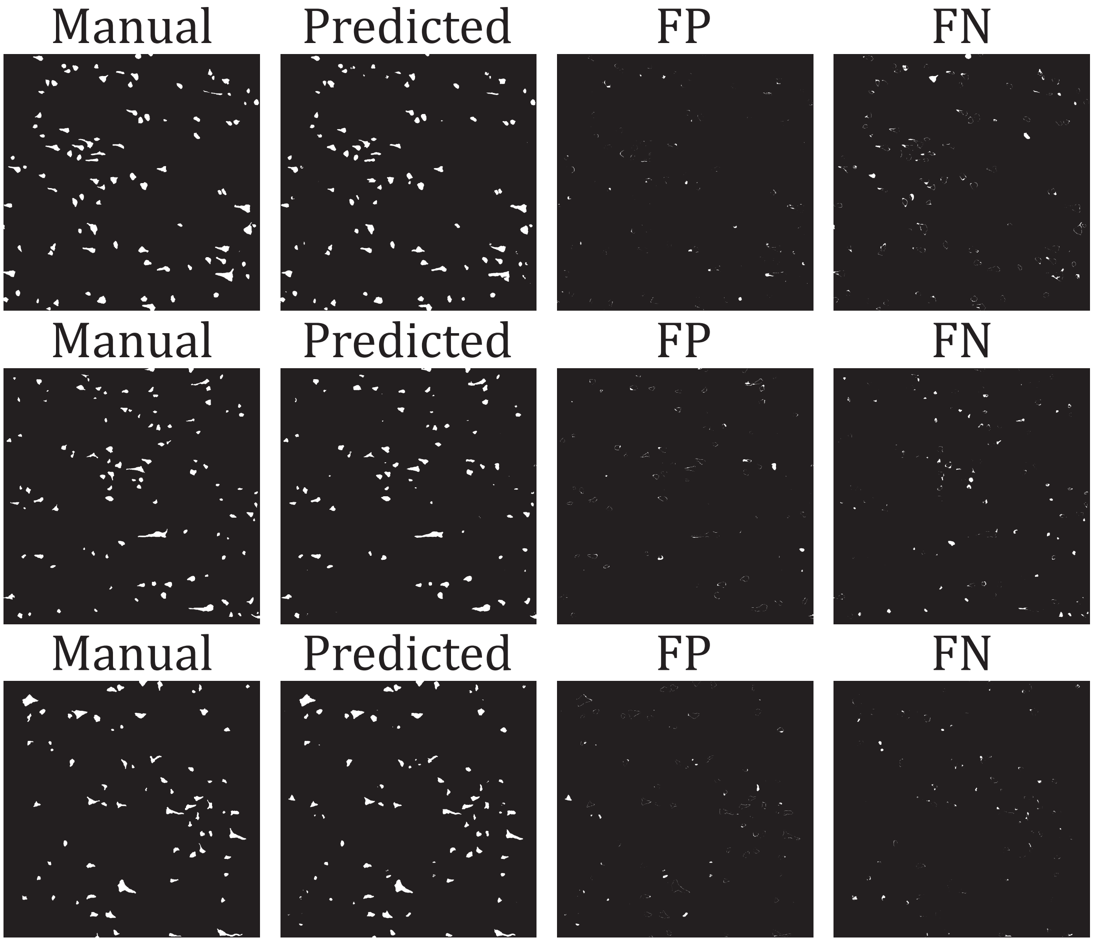

Figure S19: Test of performance of combined UNetDense model on validation images. Pyramidal cells from **Fig.S17** were manually marked by (NYL), and the same images were processed using the combined UNetDense model to predict and segment pyramidal cells. The figure shows pixels which have been measured as FP, FN, and quantitative comparisons can be found in **Table 1**. The rows correspond to Subjects 1, 2, and 3, respectively.

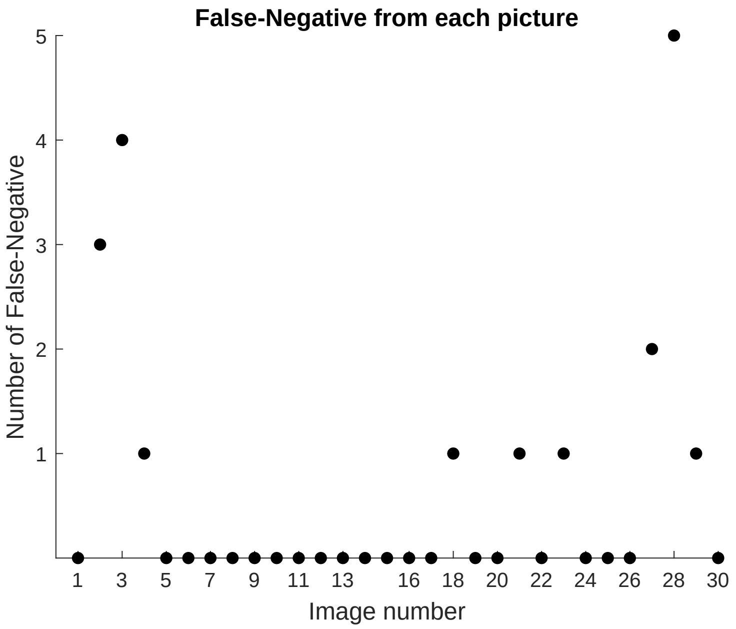

Figure S20: Detection of FN centroids from the objectwise validation. Number of FN centroid points from 30 MS images that was not detected in 30 UDP images(FN cases). The graph shows the number of FN cases in each picture.

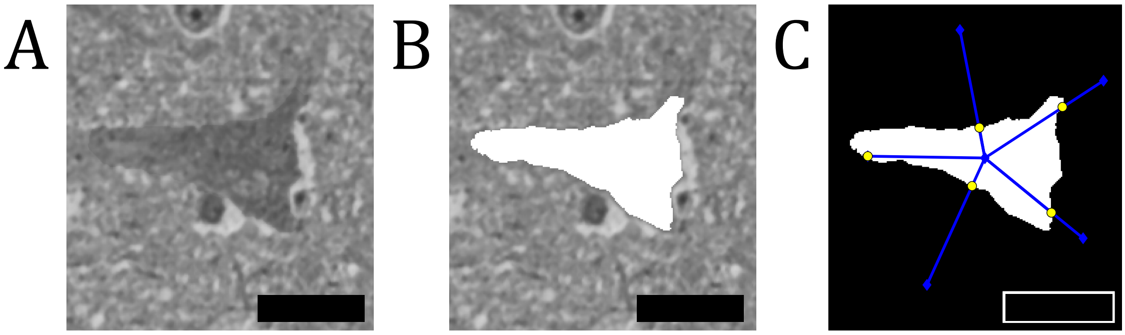

Figure S21: 2D measurement of each neuron using the nucleator. **(A)** Gray-scale image of a cell profile. Scale bar=20 μm. **(B)** Overlay image of the gray-scale image and the segmented image output from UNetDens algorithm. **(C)**The nucleator probe was applied to a cell-profile. In this case, the reference point was the centroid. A random boundary point of the cell was selected, whereas five segments with a spacing between each other of 72° (360°/5) was superimposed on top of a cell. The average line segment was then calculated to estimate the cell average radius.

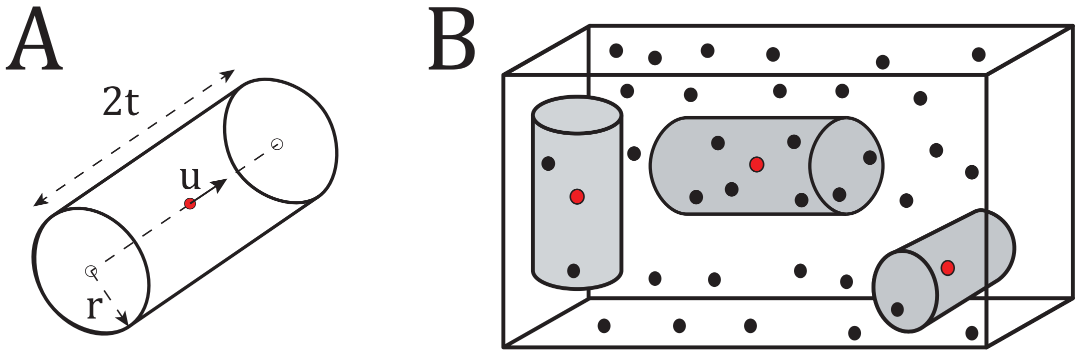

Figure S22: Illustrations of the cylinders of the cylindrical K-function. **(A)** A cylinder with radius r, height $2t$, and direction u, which is the structuring element of the cylindrical K-function. **(B)** Three different cylinders of the same size directed along the x-, y-, and z-axis, respectively, and centered at a randomly selected point of the point pattern.
